# Optimizing NTS-Polyplex as a Tool for Gene Transfer to Cultured Dopamine Neurons

**DOI:** 10.1371/journal.pone.0051341

**Published:** 2012-12-26

**Authors:** Daniel Hernandez-Baltazar, Daniel Martinez-Fong, Louis-Eric Trudeau

**Affiliations:** 1 Departamento de Fisiología, Biofísica y Neurociencias, Centro de Investigación y de Estudios Avanzados del Instituto Politécnico Nacional (CINVESTAV-IPN), México, D.F., México; 2 Department of Pharmacology, Groupe de Recherche sur le Système Nerveux Central, Faculty of Medicine, Université de Montreal, Montréal, Québec, Canada; Centre for Addiction and Mental Health, Canada

## Abstract

The study of signal transduction in dopamine (DA)-containing neurons as well as the development of new therapeutic approaches for Parkinson's disease requires the selective expression of transgenes in such neurons. Here we describe optimization of the use of the NTS-polyplex, a gene carrier system taking advantage of neurotensin receptor internalization, to transfect mouse DA neurons in primary culture. The plasmids DsRed2 (4.7 kbp) and VGLUT2-Venus (11 kbp) were used to compare the ability of this carrier system to transfect plasmids of different sizes. We examined the impact of age of the neurons (1, 3, 5 and 8 days after seeding), of culture media used during the transfection (Neurobasal with B27 vs. conditioned medium) and of three molar ratios of plasmid DNA to carrier. While the NTS-polyplex successfully transfected both plasmids in a control N1E-115 cell line, only the pDsRed2 plasmid could be transfected in primary cultured DA neurons. We achieved 20% transfection efficiency of pDsRed2 in DA neurons, with 80% cell viability. The transfection was demonstrated pharmacologically to be dependent on activation of neurotensin receptors and to be selective for DA neurons. The presence of conditioned medium for transfection was found to be required to insure cell viability. Highest transfection efficiency was achieved in the most mature neurons. In contrast, transfection with the VGLUT2-Venus plasmid produced cell damage, most likely due to the high molar ratios required, as evidenced by a 15% cell viability of DA neurons at the three molar ratios tested (1∶36, 1∶39 and 1∶42). We conclude that, when used at molar ratios lower than 1∶33, the NTS-polyplex can selectively transfect mature cultured DA neurons with only low levels of toxicity. Our results provide evidence that the NTS-polyplex has good potential for targeted gene delivery in cultured DA neurons, an *in vitro* system of great use for the screening of new therapeutic approaches for Parkinson's disease.

## Introduction

Selective gene transfer to dopamine (DA) neurons has been of much interest to study signal transduction in this cell population, which is otherwise critical for a number of physiological functions such as motor control or motivation. Since these neurons are also perturbed in diseases such as addiction, schizophrenia and Parkinson's disease, gene transfer to DA neurons has also been sought in the context of gene therapy strategies. Although selective transfection of DA neurons can be achieved using viral vectors, which can allow high rates of infection, the use of cell-specific promoters is typically required and the procedure to prepare the required constructs and to purify viral particles can be labour intensive. An alternative strategy is the use of synthetic nanoparticles that are targeted to specific cell populations through the use of cell surface receptor ligands. The neurotensin (NTS)-polyplex consists of synthetic nanoparticles that can transfer plasmid DNA (pDNA) encoding any gene of interest into cells that express and internalize the high affinity NTS receptor (NTSR1), including DA neurons. The nanoparticles result from compaction of pDNA by the electrostatic binding of a karyophilic peptide (KP) and the NTS-carrier, which is a conjugate of poly-L-lysine, NTS and a fusogenic peptide (FP) [1]. The NTS-polyplex vector promotes gene entry through endocytosis of NTSR1 [2], [3]. The transfection efficiency of NTS-polyplex is lower than that of viral vectors, but it was recently improved by coupling the vector with two short viral peptides, the hemagglutinin-derived HA2 FP and the Vp1 SV40 KP [4]. These viral peptides are thought to rescue the NTS-polyplex from acidic endosomes and to target the pDNA to the cell nucleus respectively, thus enhancing transfection efficiency and prolonging transgene expression [1], [4]. The transfection of neurotrophic factor genes such as GDNF, NTRN or BDNF, into DA neurons in a rat model of Parkinson's disease has provided support for the clinical potential of this gene transfer strategy [5], [6]. Although viral gene therapy for Parkinson's disease has been under evaluation for a number or years [7]–[9], the availability of alternate gene transfer methods remains important considering the concerns with potential immune reactions to viral vectors [10], [11] and potential oncogenicity of viral vectors able to integrate the transgene into the host genome [12], [13]. In addition, for routine gene transfer to DA neurons *in vitro*, the NTS-polyplex could be of much interest since its production is typically less labour intensive than viral vector construction.

In order to continue optimizing the NTS-polyplex vector and to further enhance its transfection efficiency, the use of DA neuron primary cultures would be of much help. However, to date, there has been no report of the use of the NTS polyplex in such a culture system. In the present work, we used a well-established postnatal mesencephalic DA neuron primary culture system [14], [15] to examine the ability of the NTS polyplex nanoparticle system to transfect DA neurons. We used the NTS-polyplex combined with two different plasmids of different molecular sizes, namely pDsRed2 (4.7 kbp) and pVGLUT2-Venus (11 kbp). We also examined the impact of age of the cultures (1, 3, 5 and 8 days after seeding), two culture media and three molar ratios of pDNA to NTS-carrier. We find that mature (8 days) DA neurons can be efficiently transfected with the NTS polyplex system, but that large plasmid size is associated with cellular toxicity.

## Methods

### Synthesis of the NTS-polyplex carrier and determination of optimal molar ratios

The detailed procedure of NTS-carrier synthesis and of NTS-polyplex formation at an optimal molar ratio and its biophysical properties have been previously reported [1], [2], [4], [16]. Briefly, NTS (Sigma Co., Saint Louis, MO) and FP (GLFEAIAEFIEGGWEGLIEGCAKKK; purity 90%; SynPep Corp., Dublin, CA) were cross-linked with poly-L-lysine (48 kDa mean molecular mass) using LC-SPDP as the cross-linker. Gel-filtration chromatography was used to purify the SPDP-derivatives and the NT-SPDP-(FP-SPDP)-poly-L-lysine conjugate, thereafter called “NTS-carrier”. This conjugate was concentrated in a volume of 1 mL, further dialyzed against phosphate-buffered saline (PBS, pH 7.4) and sterilized by filtration. Retention and retardation gel assays were used to determine the optimal molar ratio of polyplex components [4]. The retardation gel assay was performed with a constant concentration of plasmid DNA (6 nM) and increasing concentrations of KP. The retention gel assay was performed with plasmid DNA-KP complex at the optimal molar ratio (determined from the retardation gel assay) with increasing concentrations of NTS-carrier [1]. Based on previous experience, three molar ratios of plasmid to NTS-carrier were chosen for each plasmid tested: pEGFP (1∶21, 1∶24 and 1∶27), pDsRed2 (1∶27, 1∶30, and 1∶33) and pVGLUT2-Venus (1∶36, 1∶39 and 1∶42). The NTS-polyplex components for each optimal molar ratio were, for pEGFP: 6 nM pDNA:6 µM KP: 126 nM NTS-carrier (ratio of 1∶21), 6 nM pDNA:6 µM KP: 144 nM NTS-carrier (ratio of 1∶24) and 6 nM pDNA:6 µM KP: 162 nM NTS-carrier (ratio of 1∶27), for pDsRed2: 6 nM pDNA:6 µM KP: 162 nM NTS-carrier (ratio of 1∶27), 6 nM pDNA:6 µM KP: 180 nM NTS-carrier (ratio of 1∶30) and 6 nM pDNA:6 µM KP: 198 nM NTS-carrier (ratio of 1∶33), and for pVGLUT2-Venus: 6 nM pDNA:7 µM KP: 216 nM NTS-carrier (ratio of 1∶36), 6 nM pDNA:7 µM KP: 234 nM NTS-carrier (ratio of 1∶39) and 6 nM pDNA:7 µM KP: 252 nM NTS-carrier (ratio of 1∶42). Electrophoresis was carried out at 80 V for 45 min using a 0.8% agarose gels. TAE 1X was used as running buffer. The gels were stained with ethidium bromide. Gel images were acquired using Kodak EDAS 290 software.

### Plasmids

The pEGFP-N1 plasmid (4.7 kbp) codes for enhanced green-fluorescent protein (EGFP) under control of the cytomegalovirus promoter (Clontech Laboratories Inc., Mountain View, CA, USA). The pDsRed2-N1 plasmid (4.7 kbp) codes for the red fluorescent protein (RFP) under the control of cytomegalovirus promoter (Clontech). The pVGLUT2-Venus plasmid (11 kbp) codes for the type 2 vesicular glutamate transporter (VGLUT2) fused with the Venus green fluorescent protein under the control of synapsin neuron-specific promoter (kind gift of Dr. Etienne Herzog). The amplification and purification of plasmids was performed using plasmid maxi prep kits (Qiagen Inc., Toronto, Ontario, Canada) and EZ-10 purification columns (Bio Basic Inc., Markham, Ontario, Canada).

### Culture of NIE-115 cells

Wild-type N1E-115 cells (ATCC, Rockville, MD, USA) were cultured in Dulbecco's modified Eagle's medium (DMEM; MultiCell Technologies, Woonsocket, RI, USA) supplemented with 10% fetal bovine serum (Life Technologies, Burlington, Ontario, Canada) and penicillin-streptomycin (100 µg/mL of each; VWR International, Ville Mont-Royal, Quebec, Canada). Cell cultures were kept at 37°C under a 5% CO_2_ atmosphere. Addition of fresh medium was performed each 48 hours.

### Culture of DA neurons

Primary cultures of mesencephalic neurons were prepared from P0–P2 TH-EGFP/21–31 transgenic mouse pups, as previously described [14], [15]. The use of animals to obtain the primary cultures was approved by the Université de Montréal animal ethics committee (CDEA) (protocol #11–191). All efforts were made to minimize the number of animals used and their suffering. Briefly, dissociated neurons were plated on cortical astrocytes grown in monolayers on pre-coated glass coverslips. To prepare astrocyte cultures, TH-EGFP/21–31 mice pups (P0–P2) were cryoanasthetized. Cells from the cerebral cortex were enzymatically dissociated using papain (Worthington Biochemical Corp., Lakewood, NJ, USA) and were grown in culture flasks for 5–10 days in Basal Medium Eagle with added Earl's Salts (Sigma-Aldrich, Oakville, Ontario, Canada) and supplemented with penicillin/streptomycin, GlutaMAX-1 (Life Technologies), Mito+ serum extender (VWR International) and 10% fetal calf serum (Life Technologies). A cold wash with vigorous shaking was used to dislodge neurons and microglial cells after 2 days in culture. After reaching confluence, astrocytes were trypsinized, washed, collected and plated at 100 000 living cells per milliliter on collagen/poly L- lysine-coated coverslips. To prepare neurons, 1 to 2-mm thick coronal slice was cut at the level of the midbrain flexure. The ventral tegmental area and substantia nigra were isolated by microdissection. The tissue was digested with papain before being gently triturated. The dissociated cells were then collected by centrifugation and diluted at a density to optimize neuronal viability (240 000 living cells) and plated onto a pre-established astrocyte culture. Cultures were incubated at 37°C in 5% CO2 atmosphere and maintained in Neurobasal-A/B27 medium (Life Technologies) supplemented with penicillin/streptomycin, GlutaMAX-1 and 10% fetal calf serum. Astrocyte-conditioned Basal Medium Eagle was added to the standard Neurobasal-A medium in a proportion of 1∶2. In experiments carried out in serum-free culture medium, Neurobasal-A/B27 medium was supplemented with penicillin/streptomycin and GlutaMAX-1, but serum and astrocyte-conditioned medium were not added.

### Transient transfection using the NTS-polyplex

**Transfection of N1E-115 cells**: N1E-115 cells were used as positive controls for transfections due to their known ability to express NTSR1, the high affinity NTS receptor [17], [18]. N1E-115 cells (12 500 living cells) were seeded on coverslips into 24-well plates (1-mm diameter) with 500 µL of DMEM. After 48 h of incubation (50% confluence), the cells were exposed to NTS-polyplexes of different molar ratios for 24 h. After the transfection period, the medium was diluted two fold with fresh medium and incubated for an additional 48 h. Upon completion of the incubation, cells were washed once with PBS (8.1 mM Na2HPO4, 1.2 mM KH2PO4, 138 mM NaCl, 2.7 mM KCl, pH 7.4) and fixed with 4% paraformaldehyde. In the case of EGFP, protein expression was evaluated directly following fixation by epifluorescence observation. In contrast, for the other proteins of interest, fixed cells were processed for indirect immunofluorescence; for DsRed2, the protein was detected using a polyclonal anti-RFP antibody made in rabbit (1∶500, Rockland p/h, CA). For VGLUT2-Venus, an anti-VGLUT2 mouse monoclonal antibody was used (1∶1000, Millipore, CA). The primary antibodies were detected using a goat Alexa 546-coupled secondary anti-rabbit or anti-mouse antibody (1∶500) (Invitrogen Canada, Burlington, Ontario). After counterstaining with 1 µM Hoechst 33258, cells were mounted with the antiquenching medium Vectashield (Vector Laboratories; Burlingame, CA, USA).

**Transfection of cultured DA neurons**: Cultured neurons were transfected with the NTS-polyplex nanoparticle system using either the pDsRed2 (4.7 kbp) or pVGLUT2-Venus (11 kbp), two plasmids of very different molecular size. The pEGFP plasmid was not tested in neurons due to the endogenous expression of EGFP by DA neurons cultured from TH-EGFP transgenic mice. To determine the best condition for the transfection of pDsRed2 and pVGLUT2-Venus in the cultures, we examined the impact of the age of the culture (1, 3, 5 and 8 days after seeding), of culture medium used to prepare the NTS-carrier (Neurobasal+B27 vs. conditioned medium) and of the molar ratio of plasmid to NTS-carrier. Coverslips containing the neuronal cultures were transferred from their original petri dish to multi-well plates (12 wells; 1.5 cm diameter) along with some of the original conditioned medium. To prepare the NTS-polyplex nanoparticles, the single components were dissolved in corresponding medium. The pDNA was first combined with KP under conditions of vigorous shaking (1000 rpm) for 30 min. The pDNA-KP complex was next mixed with NTS-carrier. Ten seconds before the start of the transfection, the conditioned medium was quickly removed. The wells were then incubated with NTS-polyplex nanoparticles at one of the tested molar ratios. After 1 h of incubation with this complex, the original conditioned medium was quickly replaced in wells. The expression of DsRed2 or VGLUT2-Venus proteins was evaluated 96 h after transfection. The neurons were washed once with PBS and fixed with 4% paraformaldehyde for 30 min. After fixation, neurons were incubated with one or a combination of the following primary antibodies: mouse monoclonal anti-TH (1∶5000; EMD Millipore Corporation, Billerica, Massachusetts, USA), rabbit polyclonal anti-RFP (1∶500; Rockland Immunochemical Inc., Gilbertsville, PA, USA), rabbit polyclonal anti-TH (1∶5000; Millipore) or mouse monoclonal anti-VGLUT2 (1∶1000; Millipore). The secondary antibodies used were (1∶500 in each case): goat anti-mouse Alexa 488, goat anti-rabbit Alexa 546, goat anti-rabbit Alexa 488 or goat anti-mouse Alexa 546 (Invitrogen). Coverslips were mounted with Vectashield and examined immediately using epifluorescence and a Nikon Eclipse TE-200 inverted microscope. Images were acquired using a Hamamatsu Orca-II C4742-98 digital camera using ImagePro Plus software (Media Cybernetics). The fluorescence was detected at excitation/emission wavelengths of 480/535 nm (green channel), 545/610 nm (red channel) or 365/400 nm (blue channel). To confirm the specificity of transfection through NTS1-mediated endocytosis, a blocking assay with the NTS1 receptor antagonist SR48692 (100 nM) was performed; pre-incubation with the antagonist was performed 10 min before transfection; the antagonist was also present throughout the transfection procedure. The negative controls for all experiments with N1E-115 cells or cultured DA neurons were cells treated under the same conditions of transfection, but without plasmid.

### Cell counting

For NIE115 cells, the number of transfected cells was counted and reported as a proportion of the total number of cells counted with the Hoechst nuclear staining; 5 fields were counted in each coverslip. For cultured neurons, the transfection efficiency was quantified by counting the number of TH-positive DA neurons expressing the transfected protein as a function of the total number of TH-positive neurons (30 fields were counted in each coverslip). To evaluate cell viability following the transfection process, the number of TH+ neurons remaining after the transfection was compared to the number found in negative controls. For cell counting, images were acquired with a 20× objective. To evaluate cell viability, phase contrast images were acquired at 20× or 40×. All cell counting was performed in three independent experiments (*n* = 3). Digital images were visualized using Image J software.

### Statistical analysis

All values are expressed as the mean ± SEM. Differences among means were analyzed using a one-way analysis of variance (ANOVA) followed by the Newman-Keuls *post hoc* test. Statistical significance was considered when *P*<0.05. NS = not significantly different.

## Results

### Determination of optimal molar ratios

**Figure 1:**
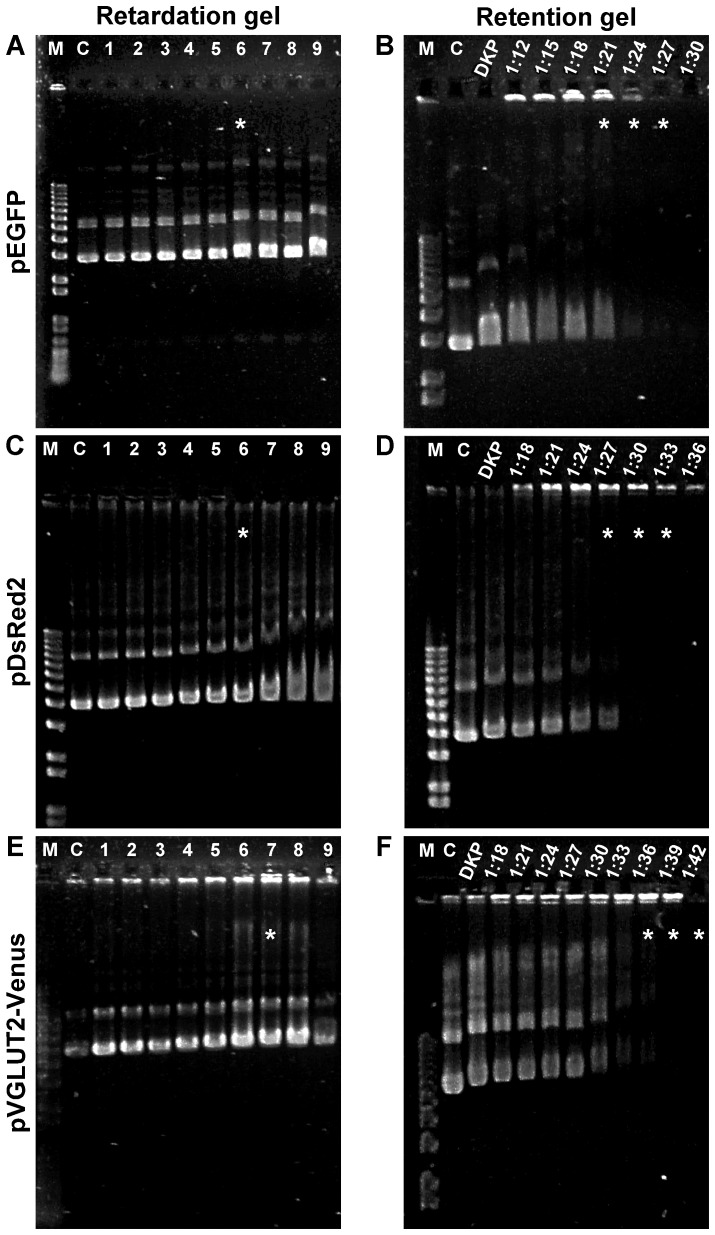
Retardation and retention gel assays. These assays were performed to determine the concentration of KP, and NTS-carrier for specific concentrations of pDNA, which results in functional NTS-polyplex nanoparticles. Each set of gels correspond to a single plasmid. In all cases, the symbol * indicates the condition(s) chosen. Electrophoresis was carried out at 80 V for 45 min on 0.8% agarose gels. TAE 1X was used as running buffer. The gels were stained with ethidium bromide. The numbers at the top of retardation gel photographs correspond to the concentration of KP in µM. M = 1 kb molecular weight markers. C = pDNA alone, DKP = pDNA-KP complex. Images were acquired using Kodak EDAS 290 software.

To optimize conditions for efficient transfection with the NTS-polyplex, we first determined the optimal molar ratio of NTS-polyplex components. For this, a retardation gel assay was performed with a constant concentration (6 nM) of plasmid DNA (pEGFP-N1, pDsRed2-N1 or pVGLUT2-Venus) and increasing concentrations of KP (µM). Based on gene expression assays, the optimal molar ratio between pDNA and KP corresponds to the first concentration of KP that retards the pDNA band over the superior border of the control pDNA band [1], [4]. Using this strategy, we found that 6 µM of KP was optimal for pEGFP-N1 (Fig. 1A) and pDsRed2 (Fig. 1C) and 7 µM for pVGLUT2-Venus (Fig. 1E). To define the optimal molar ratio of pDNA-KP complex to NTS-carrier in order to transfect N1E-115 cells and cultured DA neurons, retention gel assays were next performed with constant plasmid DNA-KP complexes (determined from the retardation gel assays) and increasing concentrations of NTS-carrier. As previously validated, the optimal molar ratio between pDNA-KP complex (formed at the optimal molar ratio) and NTS-carrier corresponds to the concentration of NTS-carrier that produces the last visible retention of the pDNA-KP complex band compared to the control well [1], [4], [16]. Seven to nine different ratios were examined and three were identified as potentially optimal based on the retention gel. For pEGFP-N1, this was 1∶21, 1∶24 and 1∶27 (Fig. 1B), for pDsRed2 this was 1∶27, 1∶30, and 1∶33 (Fig. 1D) and for pVGLUT2-Venus, this was 1∶36, 1∶39 and 1∶42 (Fig. 1F).

**Figure 2:**
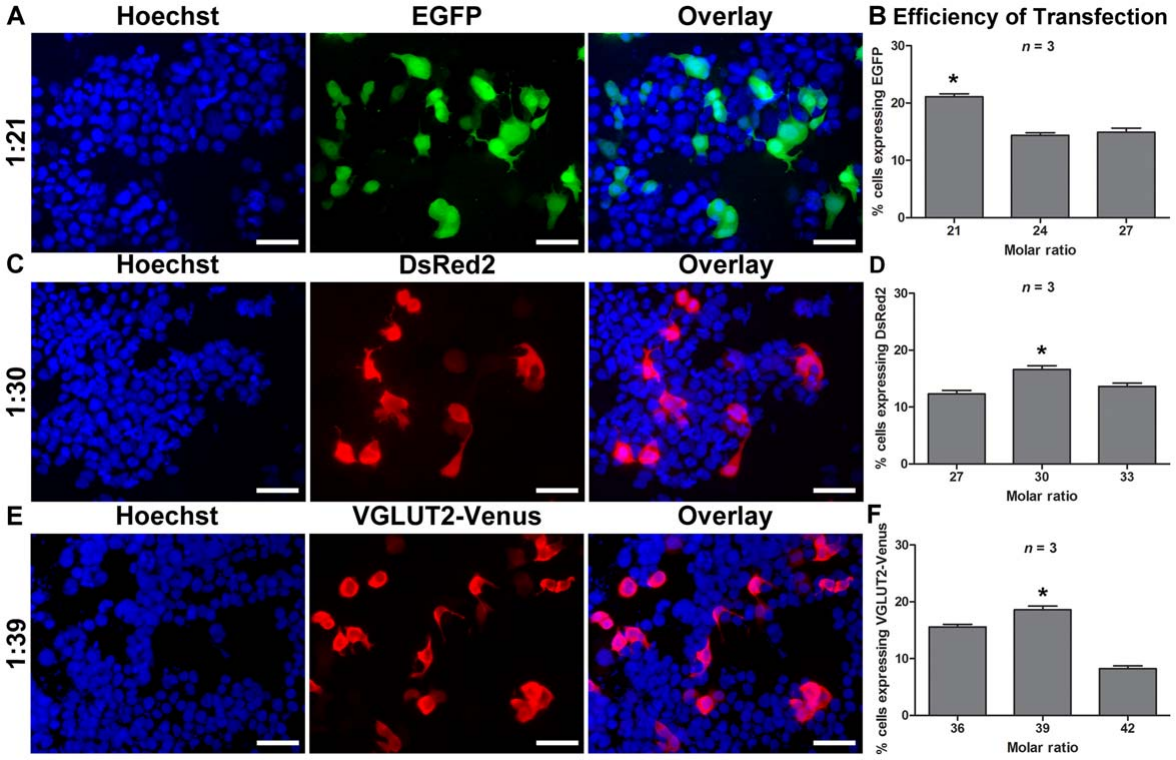
Efficiency of transfection of N1E-115 cells by the NTS-polyplex. In A, the micrographs illustrate expression of GFP (green), DsRed (red) or VGLUT2-Venus (red). Nuclei were stained with Hoechst 33258 (blue). The images were acquired using a 20× objective. In B, the graphs depict the efficiency of transfection evaluated at different plasmid to NTS-carrier molar ratios. The cell counting for each molar ratio was performed in 5 fields in each coverslip, *n* = 3. *P<0.05. Scale bar = 50 µm.

N1E-115 cells were used as positive control to verify the efficient transfection of the different plasmids used (pEGFP-N1, pDsRed2 and pVGLUT2-Venus). The expression of protein was evaluated 48 h after transfection. We found that at the optimal molar ratio, approximately 20% of N1E-115 cells expressed the GFP protein (Fig. 2A). The optimal molar ratio for pEGFP-N1 was 1∶21 (Fig. 2B). For pDsRed2 (Fig. 2C), the optimal molar ratio was 1∶30 (Fig. 2D), while for VGLUT2-Venus (Fig. 2E), the optimal ratio was 1∶39 (Fig. 2F).

### Selective transfection of cultured DA neurons by NTS-polyplex

**Figure 3:**
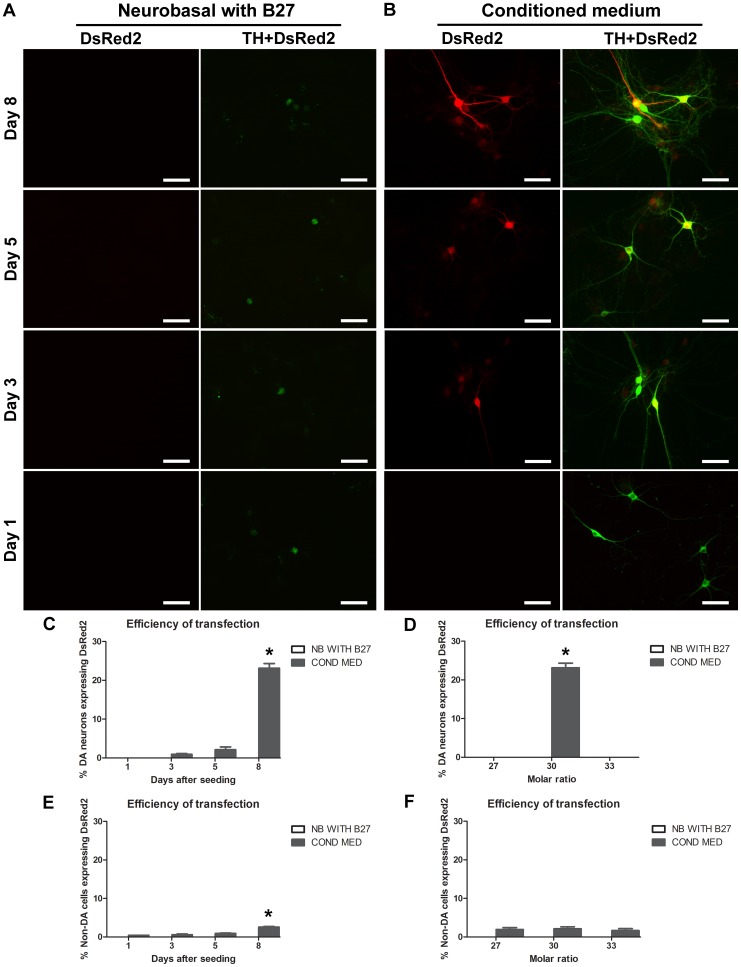
Time course and different molar ratios tested for the transient expression of pDsRed2 using NTS-polyplex. In A and B, the micrographs illustrate the appearance of TH-positive neurons and the time course of transfection of pDsRed2 with the two different culture media tested. The neurons were processed for TH (green) and DsRed (red) immunocytochemistry. The graphs in C and E correspond to the time course of transfection evaluated in DA and non-DA neurons. The graphs in D and F describe the efficiency of transfection evaluated at different plasmid to NTS-carrier molar ratios in DA and non-DA neurons (day 8 after seeding). The cell counting for each molar ratio and for the time course was performed in 30 fields per coverslip (*n* = 3 coverslips). Negative controls were also prepared using NTS-carrier without plasmid (not shown). The images were acquired with a 20× objective. *P<0.05. Scale bar = 50 µm.

**Transfection of pDsRed2**: In order to optimize the transfection of pDsRed2, we tested the efficiency of transfection at 1, 3, 5 and 8 days after seeding. For this purpose, we used the three molar ratios identified as optimal by the retention gel assay (1∶27, 1∶30 and 1∶33) (Fig. 1). Because postnatal DA neurons in primary culture are known to be highly sensitive to the specific culture medium used for their growth, we also evaluated the use of two different media during the transfection procedure: we compared a serum-free medium (Neurobasal+B27) and the complete culture medium used for the growth of the cultures (Neurobasal+B27 conditioned medium with serum). We found that when the transfection procedure was carried out in serum-free medium, both TH-positive and TH-negative neurons appeared damaged, with very few neurites (Fig. 3A) and no transfected cells could be detected (Fig. 3C–F), suggesting that the transfection procedure was cytotoxic under such conditions. In the presence of complete conditioned medium with serum, the appearance of neurons appeared relatively normal, with an abundance of elaborate dendritic and axonal-like TH-positive processes (Fig. 3B). Neurons expressing the DsRed protein could also be detected, especially in more mature neurons, transfected 8 days after seeding (Fig. 3C). A total of 23±2.17% of TH-positive neurons were transfected at 8 days, with much reduced levels found at 3 and 5 days (Fig. 3C). TH-positive DA neurons were preferentially transfected by the NTS-polyplex nanoparticles as less than 4% of 8-day-old TH-negative neurons were transfected (Fig. 3E). Strikingly, successful transfection of DA neurons was only observed at 1∶30 molar ratio (Fig. 3D). In TH-negative neurons, a negligible transfection rate (<3%) was observed at the three ratios examined (Fig. 3F).

**Figure 4:**
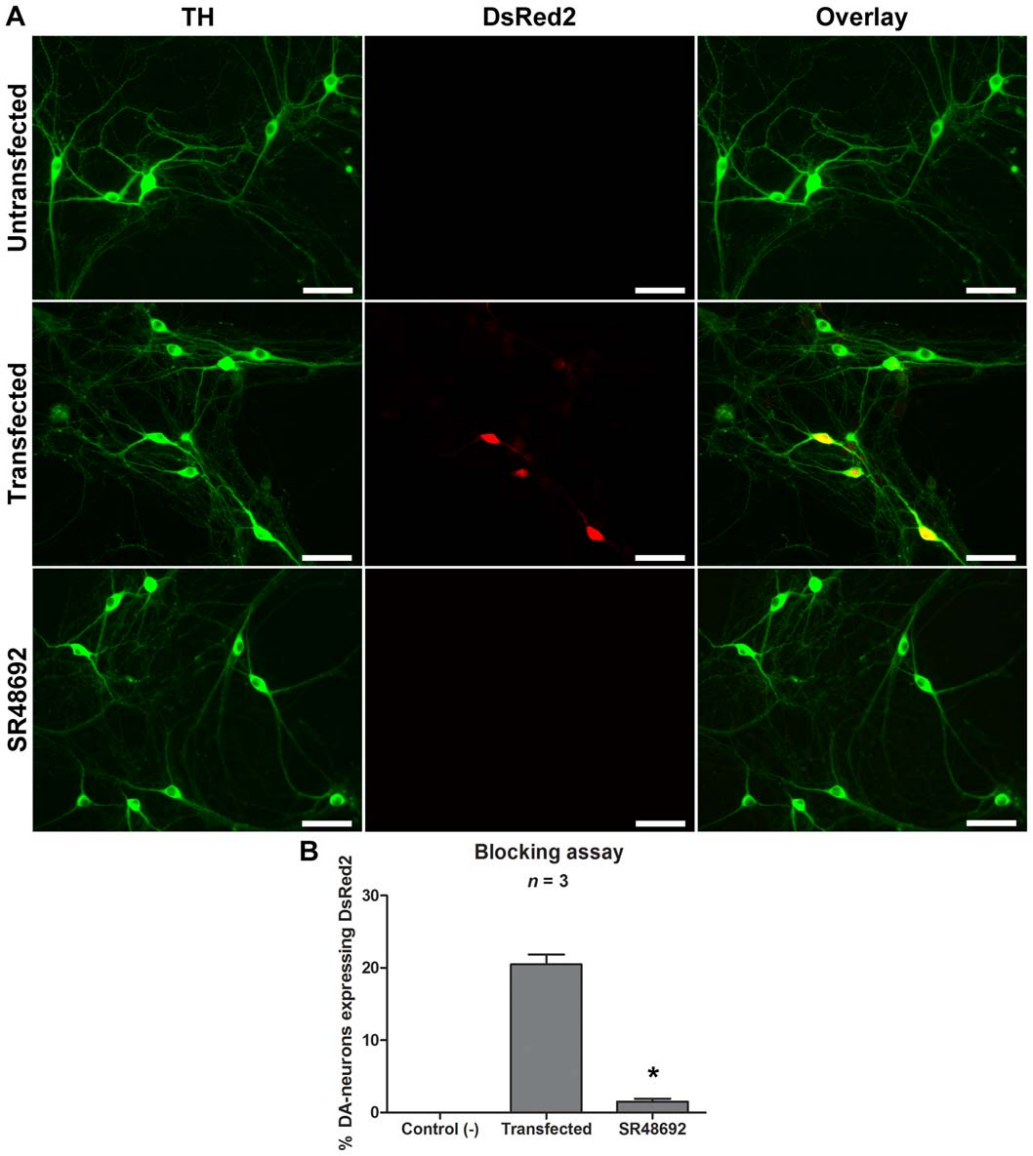
Transfection with NTS-polyplex requires NTSR1 activation. The NTSR1 antagonist SR48692 was used at a concentration of 100 nM. In panel A, the micrographs illustrate that the NTSR1-antagonist blocked pDsRed2 transfection. The neurons were processed for immunocytochemistry against TH (green) and DsRed (red). The transfections were performed at 1∶30 molar ratio in 8-day-old neurons in the presence of complete culture medium with serum. Panel B shows the efficiency of transfection for each condition. Negative controls were prepared using NTS-carrier without plasmid. The cell counting was performed from 30 fields per coverslip (*n* = 3). The images were acquired using a 20× objective. *P<0.05. Scale bar = 50 µm.

**Specificity of transfection by NTS-polyplex**: The NTS-polyplex was designed to transfect neurons taking advantage of NTSR1 internalization. To validate that such specificity was maintained in primary cultured neurons, we determined the impact of NTSR1 blockade on transfection efficiency. We found that in 8 day old cultures, a 10 min pre-incubation with 100 nM of the NTSR1 antagonist SR48692 blocked transfection with pDsRed2 (Fig. 4A,B). These results confirm that transfection with NTS-polyplex requires NTSR1 activation.

**Figure 5:**
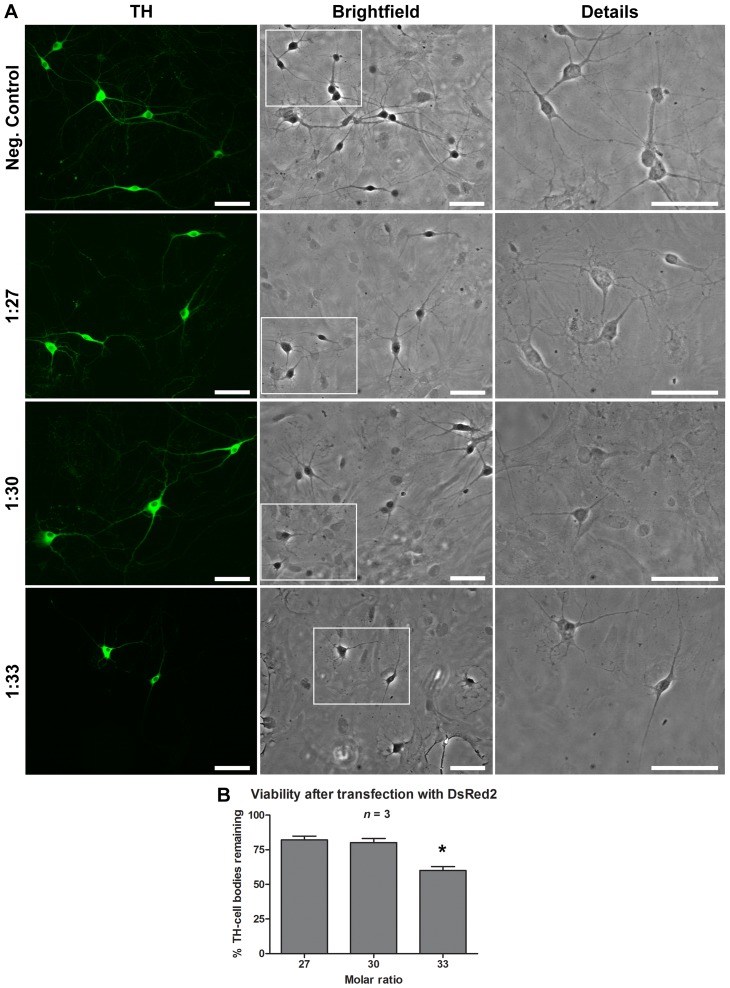
Transfection of pDsRed2 using the NTS-polyplex caused a reduction in DA neuron number only at the highest ratio tested. Panel A shows phase contrast micrographs illustrating the appearance of DA neurons after transfection using three molar ratios. In B, the graph depicts the average number of TH-positive DA neurons relative to non-transfected controls (%). The neurons were processed for TH immunocytochemistry (green). Negative controls were exposed to NTS-carrier without plasmid. The cell counting was performed from 30 fields per coverslip (*n* = 3). The images were acquired using a 20× objective. *P<0.05. Scale bar = 50 µm.

**Figure 6:**
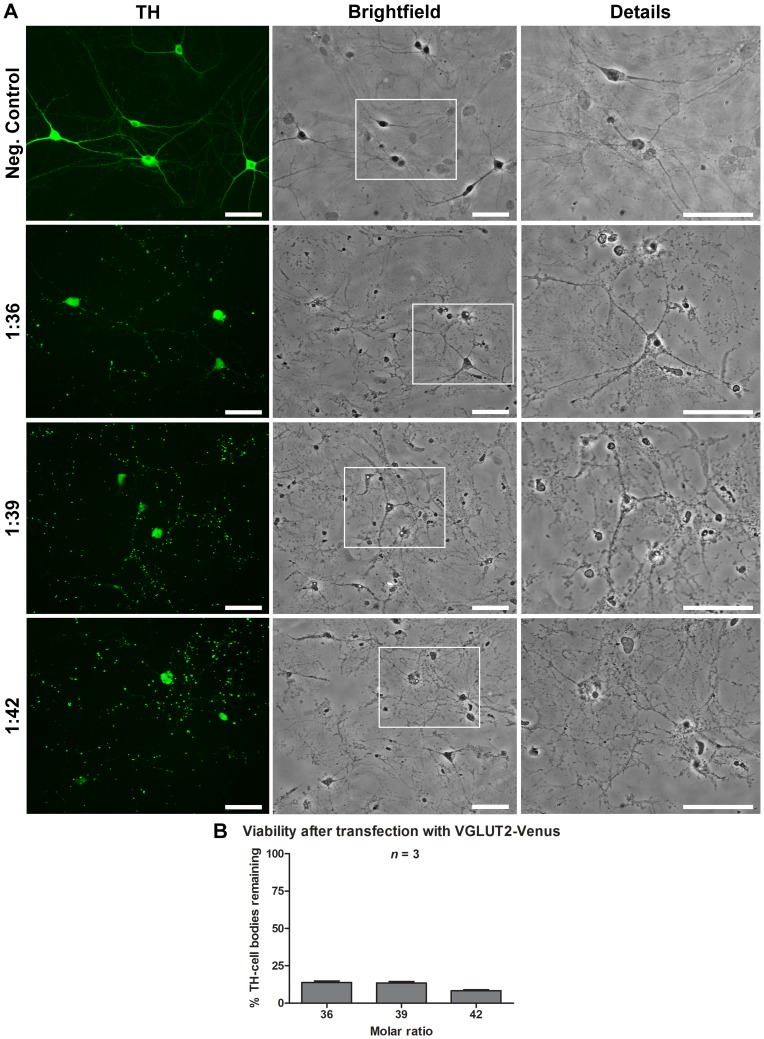
Decreased cell viability after VGLUT2-Venus transfection using the NTS-polyplex. Transfection of pVGLUT2-Venus using the NTS-polyplex caused substantial damage to both neurons and astrocytes at the three molar ratios tested. Panel A shows phase contrast micrographs illustrating the appearance of DA neurons and astrocytes after transfection using three molar ratios. In B, the graph depicts the average number of TH-positive DA neurons relative to non-transfected controls (%). The neurons were processed for TH immunocytochemistry (green). Negative controls were exposed to NTS-carrier without plasmid. The cell counting was performed from 30 fields per coverslip (*n* = 3). The images were acquired using a 20× objective. *P<0.05. Scale bar = 50 µm.

**Cell viability after pDsRed2 and pVGLUT2-venus transfection**: We noticed that although the overall appearance of neurons and astrocytes was normal at the 1∶27 and 1∶30 molar ratios (Fig. 5A), at the highest molar ratio used (1∶33), NTS-polyplex transfection with pDsRed2 lead to a reduction of 35±2.12% in the viability of TH-positive DA neurons (Fig. 5B). To further examine whether larger molar ratios of plasmid to carrier, typically required for plasmids of larger sizes, reproducibly lead to reduced neuronal viability, we next evaluated transfection with VGLUT2-Venus, a fusion protein of the green fluorescent protein Venus with the type 2 vesicular glutamate transporter. As shown previously, optimal molar ratios for this relatively large 11 kbp plasmid were 1∶36, 1∶39 and 1∶42 (Fig. 1). We found that cultures transfected with pVGLUT2-Venus showed clear signs of cell damage and death, both in neurons and in the astrocyte monolayer (Fig. 6A). This was associated with loss of more than 85% of TH-positive DA neurons (Fig. 6B). In the few remaining DA neurons, abnormal expression VGLUT2 was observed in soma and neuronal processes (results not shown).

## Discussion

Transfection of DA neurons is required to study signal transduction in this important cell population as well as to develop novel neuroprotective strategies. Although the use of viral vectors represents a powerful and efficient strategy, non-viral strategies are also of interest as alternatives considering the potential risk of immune reactions to viral vectors [10], [11] and the potential oncogenicity of retroviral vectors because of insertional activation of oncogenes [12], [13]. Although several non-viral transfection strategies have been tested in DA neurons, including calcium-phosphate co-precipitation [14], [19], [20] and various cationic liposomes and non-liposome formulations [21]–[23], in most of these methods, only low proportions of DA neurons can be transfected and selective transfection of DA neurons requires the use of cell specific promoters [20]. Through its ability to bind to NTSR1, a peptide receptor abundantly expressed in mesencephalic DA neurons [20], [24], [25], the NTS-polyplex gene transfer method facilitates selective transfection of DA neurons, as revealed by prior work, carried out mostly *in vivo* [1]–[5], [16]. However, the proportion of DA neurons transfected by this vector and the possibility to further optimize its effectiveness have not been extensively examined. In the present work, we used a primary DA neuron culture model to examine the effectiveness and selectivity of transfection of DA neurons by the NTS-polyplex system. We find that as *in vivo*, cultured postnatal DA neurons in primary culture can be transfected using the NTS-polyplex and that transfection efficiency increased with time after plating. Furthermore, we find that the transfection is highly selective for DA neurons and that transfection can only be achieved at the optimal molar ratio of pDNA to NTS-carrier. Finally, we find that for large plasmids that require a high molar ratio of NTS-carrier, neurotoxicity becomes a limiting factor.

Embryonic and postnatal DA neurons in primary culture have been extensively used to examine the physiological, biophysical and pharmacological properties of these neurons [14], [15], [26]–[29]. They have also been of great use to examine the potential of growth factors and other strategies to rescue DA neurons in Parkinson's disease models [30]–[35]. Here we used a well-described postnatal mouse primary DA neuron culture model to examine the effectiveness of the NTS-polyplex gene transfer system to transfect DA neurons. We found that at the optimal molar ratio of plasmid to carrier, we could transfect up to 20% of DA neurons. Such a proportion is similar to that recently reported *in vivo* using a serotype-2 adeno-associated viral vector [36] and is considerably higher than usually achieved using cationic lipid transfection systems or calcium-phosphate co-precipitation methods, where values below 5% are typical [19], [20] and thus our findings represent a considerable advance.

Our observation that transfection efficiency increased dramatically with time after seeding is unexplained at the present time. However, this finding is likely to be due to increased expression of the NTSR1 over time in culture, as the neurons mature. The critical importance of the NTSR1 in the efficiency of transfection is further confirmed by our finding that pre-incubation with the NTSR1 antagonist SR48692 essentially blocked transfection. Our observation that the transfection with DsRed was selective for DA neurons is also in keeping with the obligatory role of NTSR1 for transfection through the NTS-polyplex.

Our findings of toxicity of the NTS-polyplex under conditions of high molar ratios of pDNA to NTS-carrier and of the occurrence of transfection of DA neurons only at specific molar ratios highlight the importance of optimizing the transfections conditions for each pDNA to be used.

Currently, it is known that the NTS-polyplex nanoparticles must fulfill two conditions to cause efficient transfection: an adequate condensation of pDNA into a toroid structure and sufficient concentration of these structures at an optimal molar ratio [1]. In the present work we described a strategy that facilitates reaching the goal of identifying a useful range of molar ratios. We found that carrying out retardation and retention gel assays represents a simple, fast and inexpensive predictive strategy to determine working molar ratios [1]. While the retardation assay allowed identifying adequate proportions of pDNA and KP, the retention gel facilitated the identification of useful starting molar ratios of pDNA/KP/NTS-carrier complexes. The criterion used to select adequate molar ratios was a significant delay in the migration pattern of the DNA/KP/NTS-carrier complexes [1], [4]. Finally, our use of NIE115 cells as a transfection control allowed for excellent prediction of the ability of selected molar ratios to transfect cultured DA neurons: the molar ratio that was optimal for transfection of pDsRed2 in NIE115 cells was also the best (and only) ratio that allowed transfection of DA neurons.

We found that molar ratios that allowed packaging of pVGLUT2-Venus, a plasmid of large molecular size, lead to extensive death of DA neurons and to clear damage to the underlying astrocyte monolayer. Why higher molar ratios (>1∶36) lead to such strong toxicity to cultured neurons and astrocytes is presently unclear. However, this observation is compatible with previous work reporting toxicity at molar ratios higher than 1∶33 [1], perhaps due to the large size of the particles produced. The toxicity is unlikely to be due to VGLUT2 itself, as overexpression of this protein with viral vectors does not produce apparent toxicity (Trudeau, L-E, unpublished observations). We also report that the culture medium used for the transfection is critical for cell survival: transfection in serum-free medium led to extensive death of DA neurons. This observation is compatible with previous work showing that postnatal DA neurons in primary culture are exquisitely sensitive to culture conditions including the specific type and amount of serum used as well as the presence of supporting glial cells [15].

We conclude that the NTS-Polyplex can be used to efficiently and selectively transfect cultured DA neurons, in particular with plasmids of modest sizes that do not require high molar ratios of plasmid to carrier. Further optimization is now needed to identify conditions that can allow transfection with larger size plasmids or higher molar ratios. Additional optimization of transfection conditions including age of the neurons, duration of the transfection and post-transfection delay could potentially increase transfection efficiency over and above the 20% transfection rate identified here, thus further increasing the interest in using the NTS-polyplex gene transfer system for the transfer of genes to DA neurons *in vitro* and *in vivo* [3], [5], [37], [38].
